# Structure of the *E*. *coli* agmatinase, SPEB

**DOI:** 10.1371/journal.pone.0248991

**Published:** 2021-04-15

**Authors:** Iva Chitrakar, Syed Fardin Ahmed, Andrew T. Torelli, Jarrod B. French

**Affiliations:** 1 Department of Biochemistry and Cell Biology, Stony Brook University, Stony Brook, NY, United States of America; 2 Biochemistry and Structural Biology Graduate Program, Stony Brook University, Stony Brook, NY, United States of America; 3 Department of Chemistry, Ithaca College, Ithaca, NY, United States of America; 4 Chemistry Department, Stony Brook University, Stony Brook, NY, United States of America; 5 Hormel Institute, University of Minnesota, Austin, MN, United States of America; Russian Academy of Medical Sciences, RUSSIAN FEDERATION

## Abstract

Agmatine amidinohydrolase, or agmatinase, catalyzes the conversion of agmatine to putrescine and urea. This enzyme is found broadly across kingdoms of life and plays a critical role in polyamine biosynthesis and the regulation of agmatine concentrations. Here we describe the high-resolution X-ray crystal structure of the *E*. *coli* agmatinase, SPEB. The data showed a relatively high degree of pseudomerohedral twinning, was ultimately indexed in the *P*3_1_ space group and led to a final model with eighteen chains, corresponding to three full hexamers in the asymmetric unit. There was a solvent content of 38.5% and refined R/R_free_ values of 0.166/0.216. The protein has the conserved fold characteristic of the agmatine ureohydrolase family and displayed a high degree of structural similarity among individual protomers. Two distinct peaks of electron density were observed in the active site of most of the eighteen chains of SPEB. As the activity of this protein is known to be dependent upon manganese and the fold is similar to other dinuclear metallohydrolases, these peaks were modeled as manganese ions. The orientation of the conserved active site residues, in particular those amino acids that participate in binding the metal ions and a pair of acidic residues (D153 and E274 in SPEB) that play a role in catalysis, are similar to other agmatinase and arginase enzymes and is consistent with a hydrolytic mechanism that proceeds *via* a metal-activated hydroxide ion.

## Introduction

**Fig 1:**
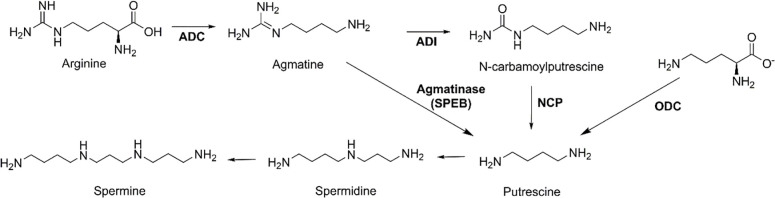
Biosynthesis of polyamines from agmatine. Putrescine can be generated from agmatine directly by an agmatinase enzyme (SPEB in *E*. *coli*), *via* two steps by the sequential action of agmatine deiminase and N-carbamoylputrescine amidohydrolase, or from ornithine by ornithine decarboxylase (ODC). Note that, although charges are not shown here for clarity, these metabolites are all expected to carry a positive charge at physiological pH.

Naturally occurring polyamines, linear cationic metabolites with multiple amino groups, are found in all kingdoms of life [1–3] and are essential for critical cellular processes [1–6]. Most organisms from bacteria to humans generate polyamines from ornithine, a product of the urea cycle, through the action of ornithine decarboxylase (ODC). Bacteria can also access polyamines *via* arginine by multiple routes (Fig 1) involving production of the key polyamine intermediate putrescine. This can occur through decarboxylation of ornithine (ODC), amidohydrolysis of N-carbamoylputrescine (NCP), or a one-step conversion of the metabolite agmatine catalyzed by agmatinase (agmatine ureohydrolase, EC 3.5.3.11). While the biosynthetic route involving agmatine is mainly utilized by bacteria, agmatinase enzymes are also present in plants as well as mammals [2, 7–9].

Interest in the role agmatine plays in mammals expanded markedly following the discovery that agmatine is an endogenous ligand for imidazoline and adrenergic receptors [10–13]. Subsequent research has revealed a diverse range of cellular functions [14] including a protective role in resisting cellular apoptosis from Ca^2+^-induced oxidative stress [15], inhibition of nitric oxide synthases [16, 17], modulation of insulin release from pancreatic cells [18], signaling [19, 20] and regulatory roles [1, 2, 21, 22], as well as an intriguing capability to induce antizyme, a negative regulator of cellular polyamine biosynthesis and cellular proliferation [23]. Agmatine is now also considered salient to a number of pathophysiological processes affecting the central nervous system [13, 24]. Cellular homeostasis of agmatine has therefore emerged as an important consideration for several avenues of research. Production of agmatine occurs by decarboxylation of arginine and is catalyzed by the activity of arginine decarboxylase [3, 25, 26]. Agmatine can be consumed by several enzymes, including agmatinase, which catalyzes the one-step conversion to putrescine (Fig 1).

Agmatinase belongs to the family of arginase enzymes [27] and exhibits the characteristic 8-stranded, parallel β-sheet sandwiched between 3 α-helices on either side. As a metallohydrolase, agmatinase is dependent on Mn^2+^ ions for catalytic activity [28], two of which are bound at the active site in high- and low-affinity sites to serve in activating a nucleophilic water molecule for catalysis [14, 28, 29]. One of the most widely studied bacterial agmatinases is the *E*. *coli* enzyme, SPEB, for which kinetic studies have been completed [29–32]. However, while the structure of putative agmatinase enzymes have been reported for several other bacterial species [33–35], no structure of *E*. coli agmatinase has yet been solved. We report here the crystal structure of agmatinase from *E*. *coli* (SPEB) in order to advance our understanding of this important enzyme and the role it plays in regulating agmatine.

## Materials and methods

### Chemicals and other reagents used

All commercially-sourced reagents were of the highest purity available and were used without further purification. Buffers and salts were sourced from Fisher BioReagents or Fisher Chemical (Fisher Scientific). Molecular biology reagents, including restriction enzymes, T4 ligase, and molecular weight markers were from New England Biolabs. Sparse matrix crystallization screens were from Hampton Research (Crystal Screens, Peg Ion Screens, and SaltRx Screens), Molecular Dimensions (Wizard screens), and Qiagen (JCSG screens). The vector used for expression of SPEB, pTHT, was a gift from Cynthia Kinsland of Cornell University.

### Gene synthesis and sub-cloning of SPEB

The *E*. *coli* agmatinase amino acid sequence (UniProt P60651) was first codon optimized for expression in an *E*. *coli* expression system using the GenSmart codon optimization tool (GenScript) (S1 Fig in S1 File). The gene was synthesized (GenScript) with a 5′ NdeI restriction endonuclease site and a stop codon followed by a XhoI restriction endonuclease site at the 3′ terminus, and delivered in a pUC57 vector (SPEB-pUC57). The gene was subcloned into the pTHT vector, a variant of pET-28 with a tobacco etch virus (TEV) protease site in place of the thrombin site. Briefly, SPEB-pUC57 and pTHT were separately digested with NdeI and XhoI (NEB). The digested gene and plasmid were separated on an agarose gel, gel purified, and then ligated using T4 DNA ligase (NEB). The ligated product was used to transform 5-alpha *E*. *coli* cells (NEB) and plated on LB agar supplemented with 50 μg/mL kanamycin. Selected colonies were cultured and the resulting plasmids (SPEB-THT) isolated for sequence verification by Sanger sequencing.

### Expression and purification of SPEB

Sequence verified SPEB-THT (S2 Fig in S1 File) was used to transform BL21(DE3) *E*. *coli* cells (NEB) and plated on LB agar supplemented with 50 μg/mL kanamycin. An overnight culture was prepared by inoculating 10 mL of LB media, supplemented with 50 μg/mL kanamycin, with a single colony of SPEB-THT in BL21 (DE3). The culture was incubated at 37°C, with vigorous shaking, for 16 hours. A large-scale culture, 1 L of LB media with 50 μg/mL kanamycin, was then inoculated with the 10 mL overnight culture. This culture was grown at 37°C, with vigorous shaking, until the OD_600_ reached 0.6. The temperature was reduced to 18°C and the culture was allowed to continue shaking for an additional 1 hour. Protein expression was induced by the addition of isopropyl β-D-1-thiogalactopyranoside (IPTG) to a final concentration of 0.1 mM. The induced culture was further incubated, with vigorous shaking, for an additional 16 hours. The cells were then harvested by centrifugation at 6,000g at 4°C for 20 minutes. The pellet was resuspended in 35 mL of lysis buffer (50 mM sodium phosphate, pH 7.4, 300 mM sodium chloride, 10 mM imidazole and 10% glycerol) and lysed by sonication. The lysate was cleared by centrifugation at 25,000 rcf at 4°C for 60 min. The protein was purified from cleared lysate by immobilized metal affinity chromatography using Ni-NTA resin (Qiagen) followed by size exclusion chromatography on an AKTA Pure system (GE). Briefly, the protein was loaded onto a pre-equilibrated 1.0 mL Ni-NTA Superflow cartridge (Qiagen), washed successively with 40 mL of lysis buffer then 20 mL of wash buffer (lysis buffer supplemented with a final concentration of 50 mM imidazole) before being eluted with 15 mL of elution buffer (lysis buffer with a final concentration of 300 mM imidazole). The elution fractions were pooled and further purified by size exclusion chromatography on a Superdex 200 10/300 GL column (GE Life Sciences) running 30 mM Tris, pH 7.0 and 50 mM sodium chloride. Fractions containing pure (>95% by SDS PAGE, S3 Fig in S1 File) protein were pooled, exchanged into buffer containing 10 mM Tris, pH 7.4, and 30 mM NaCl, concentrated to 45 mg/mL, and flash frozen in liquid nitrogen for storage at -80°C.

### Crystallization and data collection

Initial crystallization screens were conducted with commercial sparse matrix screen kits Crystal Screen I and II, Peg Ion I and II, and SaltRx I and II (Hampton Research) using hanging drop vapor diffusion. The trials were set up using 1.5 μL of protein (45 mg/mL in 10 mM Tris, pH 7.4, and 30 mM NaCl) and 1.5 μL of well solution and trays were stored at 18°C. After 4–8 days incubation time, crystals appeared in 5 separate conditions. Crystal growth conditions included: 1.0 M ammonium citrate tribasic, and 0.1 M bis-Tris propane, pH 7.0; 0.7 M sodium citrate tribasic dihydrate, and 0.1 M Bis-Tris propane, pH 7.0; 0.7 M sodium citrate tribasic dihydrate, and 0,1 M Tris, pH 8.25; 1.2 M sodium citrate tribasic dehydrate, and 0.1 M Bis-Tris propane, pH 7.0; 3.5 M sodium formate and 0.1 M Tris, pH 8.5. The resulting crystals were rod-shaped in all cases. Crystals from the latter condition were harvested and crushed for seeding using a seed bead (MiTeGen). With microseeding, diffraction-quality crystals grew in overnight in 3.5 M sodium formate and 0.1 M Tris, pH 8.5, using 24 mg/mL of SPEB protein. Crystals were flash frozen in liquid nitrogen without addition of further cryoprotectants, and stored in liquid nitrogen for data collection. Data were collected at 100 K at the North Eastern Collaborative Access team (NE-CAT) beamline, 24-ID-C, at the Advanced Photon Source, using a Pilatus 6M detector. Data collection statistics are provided in Table 1.

**Table 1:** Data collection and refinement statistics.

**Data Collection**^a^	
PDB ID	7LBA
Beamline	NE-CAT 24-ID-C
Resolution range (Å)	2.20–82.0
Wavelength (Å)	0.97918
Space Group	*P*3_1_
Unit Cell Dimensions	
a, b, c (Å)	a = b = 139.81, c = 222.99
α, β, γ	α = β = 90°; γ = 120°
Measured reflections	1,457,187
Unique reflections	247,407
Mean I/σ	11.2 (3.4)
Completeness	99.9 (100)
Redundancy	5.8 (5.9)
R_merge_ (%)	11.3 (55.7)
CC_1/2_	0.99 (0.79)
**Data Refinement**	
Resolution Range (Å)	2.20–82.0 (2.20–2.26)
Total reflections	230,595
Test set	12,411
*R*_work_	17.9
*R*_free_	21.3
No. of protein atoms	42,227
No. of metal atoms	27
No. of water atoms	1,248
RMSD from ideal	
Bonds (Å)	0.002
Angles (°)	1.21
Mean B factor (Å^2^)	26.4
Ramachandran	
Favored (%)	96.07
Additionally allowed (%)	3.85
Outliers (%)	0.07
Clashscore^b^	3.42 (99)

^a^ Numbers in parentheses correspond to values for the highest resolution shell.

^b^ Value calculated by MolProbity–value in parentheses corresponds to percentile (100% is best) when compared to a representative set of structures of comparable resolution.

### Structure solution, refinement and model building

Data were initially indexed, integrated and scaled using the automated processing pipeline RAPD at the North Eastern Collaborative Access Team (NE-CAT) facility. RAPD is a modular package of programs that automates initial data processing using XDS (to integrate and scale the data, and then uses the CCP4 [36] programs Pointless [37] and Scala [37] to analyze the data. After identifying pseudosymmetry and twinning, the data were reprocessed using Mosflm [38], Pointless [37], Aimless/Scala [37] and Ctruncate [39, 40] to index, integrate, and merge in a lower symmetry space group (see Results and Discussion for details). Molecular replacement was conducted using MolRep [41] and completed in two stages. The first stage used the default parameters with the structure of *Burkholderia thailandensis* hypothetical agmatinase (4DZ4) [34] as the search model and identified a partial solution containing 2 complete SPEB hexamers (12 total SPEB protomers). A round of restrained refinement was carried out using REFMAC5 [42] to generate initial maps. The second stage of molecular replacement, also with MolRep, used the fast rotation function and a phased translation function using structure factors from the masked map of the partial solution. This step enabled the placement of a third SPEB hexamer for a total of 18 protomers. The final model was built through iterative rounds of restrained refinement with intensity-based twin refinement, using REFMAC5, and manual model building using Coot [43]. Non-crystallographic symmetry (NCS) restraints were used during early rounds of refinement and removed for later rounds. Water molecules were added once the refinement converged. Data collection and refinement statistics are provided in Table 1.

### Structure analysis

Sequence alignments were completed using the BLAST-P suite [44] or Clustal Omega [45]. The reported sequence identity and conservation were from standard protein BLAST (BLASTP) against the Protein Data Bank proteins database. The protein structure search was completed using the Dali server [46] using the refined structure of SPEB. All known structures with >10% sequence identity, using the PDB25 subset, were reported. To calculate surface areas and free energies for the assembly, the PDBePISA server [47] was used with the refined structure of SPEB. Structural superpositions were generated using the global fit in PyMol (Schrodinger). To place the ligand in the SPEB active site, the structure of the *D*. *radiodurans* agmatinase with bound hexane-1,6-diamine (PDB code 1WOG) [33] was first superimposed on the refined SPEB structure. The agmatine ligand (PDB code AG2) was then superimposed on the 1WOG ligand as a starting point. The ligand position was then adjusted so that the two nitrogen atoms of the amidino group of agmatine were placed on the terminal nitrogen atom of the hexane-1,6-diamine ligand and a Mn-bound water atom, respectively, while the terminal amino group of agmatine was positioned over the other terminal amino group of hexane-1,6-diamine. The agmatine ligand was then regularized according to the restraints for the AG2 ligand (agmatine) from the protein data bank (PDB) definition (CIF) file. Figures were generated using PyMol v 2.3.3 (Schrodinger) and ChemDraw Prime v 16.0 (PerkinElmer). The sequence alignment was carried out using Clustal Omega [45] and the alignment figure was generated with ESPript 3 [48].

## Results and discussion

### Expression, purification, crystallization

The *E*. *coli* agmatine ureohydrolase (agmatinase, SPEB) is a 306 residue protein responsible for converting agmatine to putrescine. To define the structural basis for enzyme activity and to complement the existing body of published kinetic and functional characterization of *E*. *coli a*gmatinase, we set out to solve the X-ray crystal structure of SPEB. The protein was expressed as an N-terminally hexahistidine-tagged construct from a codon-optimized gene. The protein expressed well in LB media, yielding 10–12 mg of purified agmatinase protein per L of media. The protein was purified in two steps using conventional immobilized metal affinity chromatography with Ni-NTA media followed by size exclusion chromatography (SEC) on a Superdex 200 10/300 GL column (GE). The elution profile from SEC suggested that SPEB was a hexamer, as expected (S3 Fig in S1 File). After purification, the protein was estimated to be greater than 90% pure by SDS-PAGE (S4 Fig in S1 File). Prior to initiating crystallization trials, the protein was exchanged into a minimal buffer and concentrated to 45 mg/mL. The initial crystallization trials were conducted by hanging drop vapor diffusion using commercially available sparse matrix screening kits. Within one week, rod-shaped SPEB crystals appeared in several conditions, all containing either sodium formate or sodium citrate. Microseeding at a lower protein concentration was used to generate diffraction-quality crystals of sufficient size for data collection. Crystals for data collection were grown in 3.5 M sodium formate and 0.1 M Tris, pH 8.5, and were used without any additional cryoprotectant added. The data collection statistics are provided in Table 1.

### Structure solution

Several complete data sets were collected for SPEB crystals with diffraction visible to 2 Å or better. An inspection of the diffraction patterns revealed a relatively large dimension of the unit cell in one direction and a moderate-high level of mosaicity. The data were initially processed using the RAPD pipeline, which conducts automatic processing with XDS, Pointless and Aimless/Scala. All data sets indexed in a hexagonal space group by pointless, and a number of hexagonal space groups were analyzed. A first round of molecular replacement (MR) was run, using MolRep with the structure of the *B*. *thailandensis* hypothetical agmatinase (4DZ4) [34] as a search model, to identify the most probable space group candidate from the Laue class. A likely MR solution was found in the *P*6_1_ space group (unit cell dimensions a = b = 139.6 Å, c = 222.6 Å, α = β = 90, γ = 120), where one complete hexamer of SPEB was placed. Iterative rounds of refinement and manual model building, using REFMAC5 and Coot, respectively, improved the model, however the R_free_ value plateaued at approximately 30%. Analysis of the solvent content yielded a Matthews coefficient of 2.98 and solvent content of 58.8% for a single hexamer (6 chains) of SPEB. The predicted number of chains by analysis of the asymmetric unit (Matthews_Coeff, CCP4) was 8, with an expected solvent content of 45%. Inspection of the maps also revealed a large region of electron density that was unaccounted for, suggestive of the presence of additional chains in the asymmetric unit. Additional rounds of MR were attempted using the SPEB hexamer, protomer and different oligomeric variants. All attempts to place additional chains of SPEB, however, failed.

An inspection of the processed data for obvious pathologies was then conducted. A common warning sign for twinning is when the space group appears to be hexagonal, but is, in fact, a lower space group that is twinned. From twinning tests [39, 49, 50], it was determined that the SPEB dataset showed that the data were likely twinned (pseudomerohedral) with a twin fraction of 0.12–0.13 from the L-test and 0.41 from the H-test. Re-refinement of the *P*6_1_ data using intensity-based twin refinement improved the R-factors but MR still failed to yield a solution for the remaining density. As the twinning analysis suggested that the Laue symmetry may be too high, the data were reprocessed in several lower space groups using iMosflm [38]. A partial solution, in the *P*3_1_ space group with two complete hexamers in the asymmetric unit, was identified by MR using the partially refined SPEB structure. In the *P*3_1_ space group Matthews analysis predicted 12–18 molecules per asymmetric unit with 38–59% solvent content. As with the *P*6_1_ data set, attempts to fit additional chains using the conventional search approach failed. To identify the location of the additional chains of SPEB, we used an MR protocol in MolRep [41] consisting of using the fast rotation function against modified structure factors followed by a phased translation function. This approach, using the partially refined SPEB hexamer, yielded a clear solution with three complete SPEB hexamers in the asymmetric unit. Refinement of this solution using intensity-based twin refinement yielded high quality maps with clear density for all three SPEB hexamers. The final model, with 18 chains in the asymmetric unit, had a Matthews number of 2.0, 38.5% solvent content, and converged at R-work and R-free values of 17.9 and 21.3%, respectively. The data refinement statistics are given in Table 1. The electron density maps (S5 Fig in S1 File) were of sufficient quality to build 5,479 residues in the 18 chains of the model and to unambiguously place 27 of 36 metal ions and 1,248 water molecules. From prior studies of SPEB enzyme kinetics [14, 28, 29] and the analysis of our structural data, we expect that all of the metal binding sites are fully occupied by metal ions and are most likely to be occupied by manganese. Weaker density in parts of the third hexamer (MNOPQR), however, made placement of some of the metal atoms difficult. As such, we only placed metals in our model for those sites with definitive electron density (S5 Fig in S1 File).

### Overall structure of SPEB

**Fig 2:**
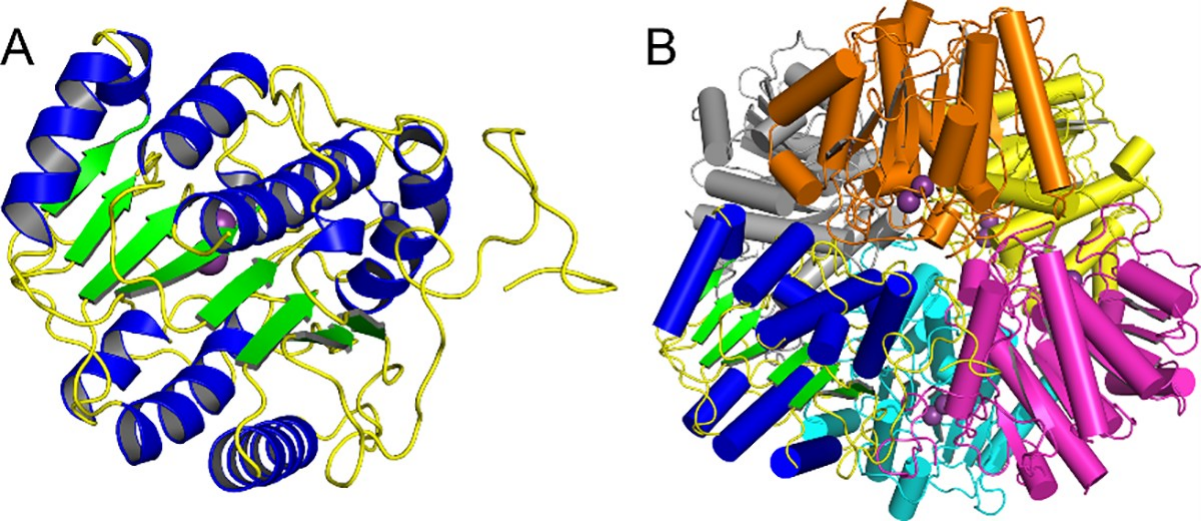
Overall structure of SPEB. The protomer of SPEB (A) shows the conserved α/β/α sandwich that is characteristic of the agmatine ureohydrolase family. The presumed biologically relevant unit, a hexamer with D3 symmetry (B), was observed in the crystal structure and was consistent with SEC data (S3 Fig in S1 File) and the prediction by PDBePISA (47).

The protomer of SPEB comprises an α/β/α sandwich with an eight-stranded parallel β-sheet between two sets of three α-helices (Fig 2A). An additional α-helix runs roughly perpendicular to the plane of the sandwich structure. The strands of the central β-sheet are organized in the order β_2_- β_1_- β_3_- β_8_- β_7_- β_4_- β_5_- β_6_. This same fold is conserved within the agmatine ureohydrolase family and, more generally, within the arginase_HDAC superfamily [27]. These proteins are all metal-dependent enzymes that catalyze hydrolysis of an amide bond. A structure similarity search of the PDB25 subset using DALI [51] yielded several members of the arginase superfamily, including agmatinase, arginase and HDAC proteins (Table 2). As anticipated, SPEB showed the highest level of structural similarity to the hypothetical agmatinase from *Burkholderia thailandensis* (PDB 4DZ4), solved as part of a structural genomics pipeline [34], with an RMSD of 1.5 Å.

**Table 2:** Dali protein structure comparison results (protein matches to PDB25 with >10% identity).

PDB ID	RMSD	% ID	Num. res.	Description
4DZ4	1.5	48	317	Hypothetical agmatinase
1XFK	2.4	21	324	Formimidoylglutamase
4Q3V	2.5	23	340	Arginase
4RHI	2.3	22	316	Arginase
4G3H	2.8	17	310	Arginase (ROCF)
6ODC	3.3	11	367	Histone deacetylase 8
6HRQ	3.4	10	419	Histone deacetylase
5EDU	3.6	10	723	Histone deac./maltose-binding periplasmic protein

**Fig 3:**
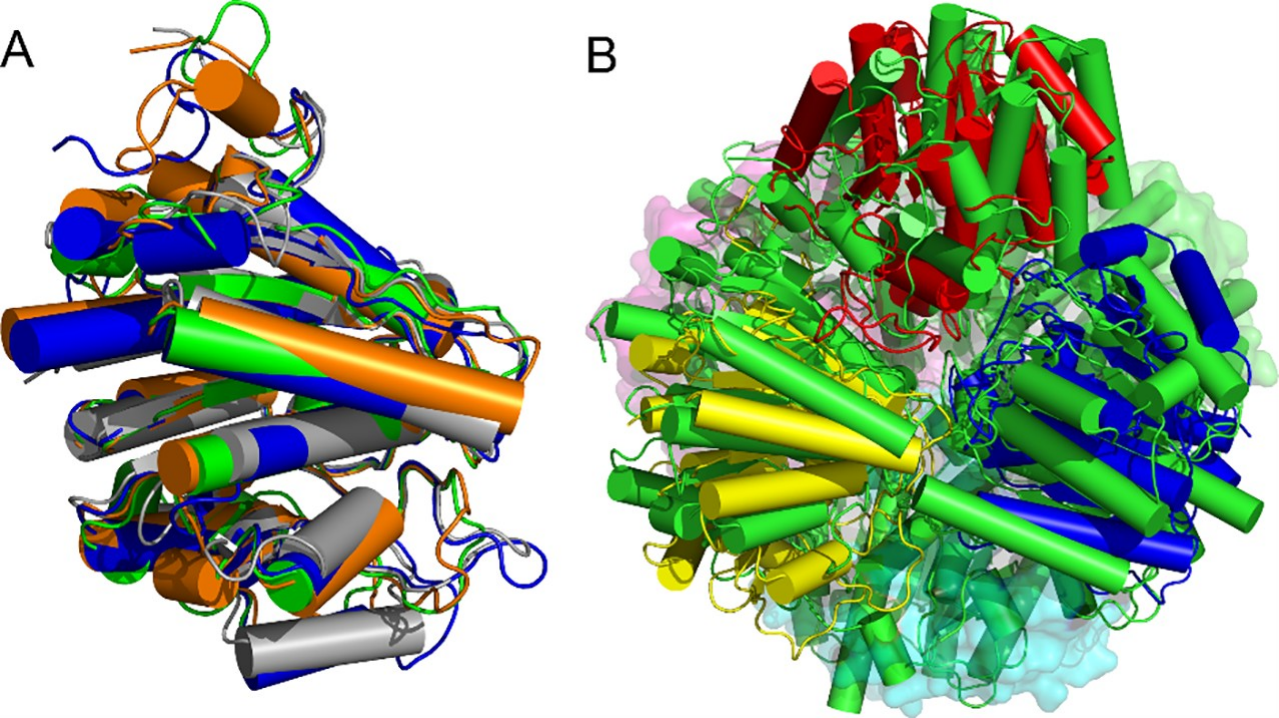
Comparison of SPEB to other arginase family proteins. The protomer of SPEB (A, green) superimposes well with the putative agmatinase from *B*. *thailandensis* (4DZ4, orange; RMSD 0.555 Å), the guanidinopropionase from *P*. *aeruginosa* (3NIP, blue; RMSD 0.793 Å), and the agmatinase from *D*. *radiodurans* (1WOG, gray; RMSD 1.13 Å). While the overall orientation of the protomers in the hexamer is similar, the structural similarity is much lower for the hexamers with an RMSD of 37.7 Å between SPEB and 4DZ4 (B, SPEB in yellow, red and blue helices for top 3 chains, bottom 3 chains shown in cyan, green and purple surface representation; 4DZ4 in green helices, see S6 Fig in S1 File for superposition with 3NIP and 1WOG).

As expected from SEC (S3 Fig in S1 File), the crystal structure revealed a clear hexameric structure of SPEB (Fig 2B). The hexamer has D3 symmetry, with very little structural variation between protomers. The RMSD for an all-atom superposition of the SPEB protomers onto one another range from 0.162 to 0.181 Å. While eukaryotic proteins of the arginase family are typically trimeric [52], their bacterial counterparts tend to be hexameric [33, 35]. Superposition of SPEB with the hypothetic agmatinase from *B*. *thailandensis* (4DZ4), *Pseudomonas aeruginosa* guanidinopropionase (3NIP), and *Deinococcus radiodurans* agmatinase (1WOG) show a high degree of conservation in the protomer structure (Fig 3A). However, while there is a relatively conserved hexameric arrangement of the protomers, the overall conservation of structure is much worse in the hexamer with RMSD values ranging from 29.0 to 37.6 Å (Fig 3B; S6 Fig in S1 File). These large RMSD values arise due to slight differences in inter-subunit distances that lead to overall poor overlap of individual atoms when the full hexamers are aligned. Superposition of the structure of SPEB with the eukaryotic agmatinase from *Schistosoma mansoni* (4Q3V) shows a reasonably well-conserved fold (S7A Fig in S1 File, RMSD of 1.4 Å for the protomer) and a similar 3-fold symmetric arrangement of the chains (S7B Fig in S1 File, RMSD of 4.6 Å for the trimer), however the orientation of the protomers, with respect to one another, is different than that of SPEB.

For the structure of SPEB, as solved in the *P*3_1_ space group, there were 3 complete hexamers in the asymmetric unit (S8 Fig in S1 File). As expected, the third hexamer added to the model after accounting for twinning (Chains M, N, O, P, Q and R) had the poorest fit, showed the highest B-factors, and had the largest number of real-space R-value Z-score (RSRZ) outliers. Overall, however, the three hexamers superimposed well, giving RMSD values that ranged from 0.165 to 0.238 Å, with the higher values for hexamer MNOPQR. Analysis of the SPEB assembly by PDBePISA [47] predicted a hexamer, as expected. The calculated surface area of the SPEB hexamer was 47,870 Å^2^ while the buried surface area was 26,250 Å^2^. The total calculated solvent free energy gain (Δ*G*^*int*^) upon formation of the hexameric assembly was -244.5 kcal/mol. Each molecule of SPEB makes surface contacts with four other protomers (Fig 2B). The average interface area, per protomer of SPEB, was 1,329.5 Å^2^, and the predicted average Δ*G*^*int*^ was -21.7 kcal/mol.

### Metal binding site and active site of SPEB

**Fig 4:**
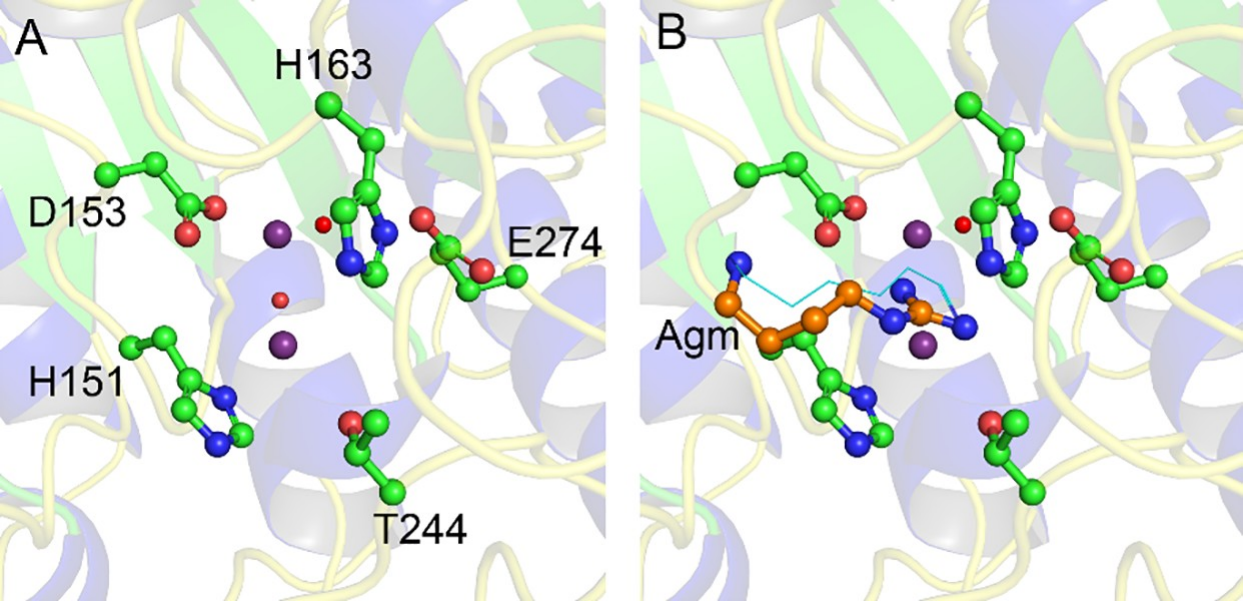
Active site of SPEB. The conserved active site residues of SPEB (A, chain A) are depicted in ball and stick representation with carbon atoms colored green, oxygen atoms colored red, and nitrogen atoms colored blue. Mn^2+^ ions are shown as purple spheres, while two active site water molecules are included as red spheres. A putative model of agmatine binding to SPEB (B, depicted with carbon atoms colored orange and nitrogen atoms colored blue) was generated by superposition to the hexane-1,6-diamine ligand in 1WOG (B, cyan line drawing, see structure analysis in Materials and Methods). The position of agmatine illustrates that protein-ligand binding is likely governed predominantly by polar contacts with the guanidinium group. Refer to Fig 5B and 5C for details about hydrogen bonding pattern and distances in the active site.

**Fig 5:**
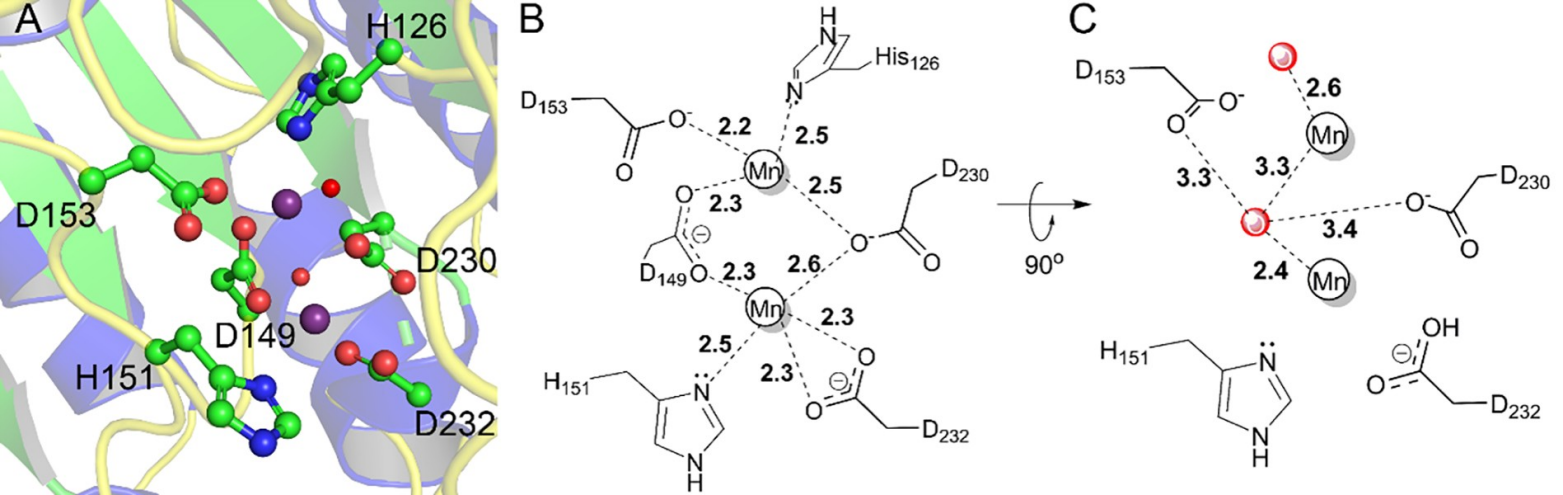
Metal binding in the SPEB active site. Two clear peaks of electron density were observed in the structure and were modeled as Mn^2+^ ions (A, represented as purple spheres). Also shown are well-defined water molecules that may play a role in catalysis (represented as red spheres). A schematic (B) shows the coordination of the metal ions by active site residues, and the corresponding bond distances. The relative positions, and distances, of the water molecules (red spheres) is similarly depicted in a schematic (C) of the same site that was rotated 90°, relative to B, as shown.

In the first two hexamers of SPEB that were built into the model (ABCDEF and GHIJKL), a pair of unambiguous spheres of electron density, presumed to be metal ions, were visible in each of the chains (S5 Fig in S1 File). The last hexamer (MNOPQR) also showed likely metal ions at these sites in each protomer, but the density was not as clear. A pair of manganese ions (Mn^2+^) is a conserved feature of this class of enzyme, and is proposed to activate a water molecule for nucleophilic attack of the substrate [14, 28, 29, 53, 54]. Superposition of SPEB with homologous agmatinase/arginase enzymes showed that the conserved Mn^2+^ ions in the homologues superimposed well with the observed spheres of electron density in the SPEB structure. Manganese has been shown to be essential for catalytic activity of SPEB [14, 28, 29]. As such, these regions were modeled as a dinuclear Mn^2+^ site in the structure of SPEB. The metal ions are octahedrally coordinated by a pair of histidine residues (H126 and H151), and four aspartate residues (D149, D153, D230, and D232) (Figs 4 and 5; S5 Fig in S1 File). Two water molecules are also clearly visible occupying the remaining coordination sites (Fig 5; S5 Fig in S1 File). The metal binding residues are a highly conserved feature of this class of enzymes, speaking to the presumed functional importance of these metals (S9 Fig in S1 File) [48].

Since the arginase_HDAC superfamily catalyzes metal-dependent hydrolysis reactions, the position of the metal(s) in the protein marks the position of the active site. We identified the active site of SPEB using the metal positions in SPEB, and by superposition with the structure of homologous arginases. We then superimposed the ligand, agmatine, onto the hexane-1,6-diamine inhibitor present in the structure of the *D*. *radiodurans* agmatinase structure (Fig 4B) [33]. Note that the position of the ligand shown in Fig 4B is not based on experimental results, but is derived solely from superposition with the *D*. *radiodurans* structure. From this analysis, it appears that the ligand makes important polar contacts with two of the amino acid side chains that coordinate the metals (H151 and D153) as well as with conserved threonine (T244), glutamate (E274), and histidine (H163) residues (Fig 5; S8 Fig in S1 File). The H163, E274, and T244 sidechains are all proposed to interact with the guanidinium group of the substrate [31, 33].

Metallohydrolases with two metal ions catalyze a diverse range of reactions and make use of several distinct enzyme mechanisms [54]. While there is, as yet, no definitive description of the SPEB mechanism, based on the conserved active site and metal-binding residues, the mechanism of SPEB is expected to be similar to that of the structurally similar-arginase enzyme [28–33, 53, 55, 56]. In this mechanism, an activated water molecule attacks the carbon of the guanidinium group, yielding a tetrahedral intermediate [53, 56]. In the case of SPEB, the substrate would likely interact with D153, T244, and E274 predominantly through polar contacts with the guanidinium group (Fig 4B), and the substrate would not directly interact with the metal ions. The activated water, coordinated between the metal ions (Fig 5C), would attack the carbon of the guanidinium group, forming a tetrahedral intermediate. After a proton transfer to the leaving amino group, the carbonyl of urea forms while the electrons of the carbon-nitrogen bond are pushed to the terminal nitrogen of what becomes the putrescine product. Similar to the proposed arginase mechanism, an aspartate-glutamate pair (D153 and E274 in SPEB, Fig 4) play important roles in properly positioning the substrate, coordinating to the metal ions, and participating in the proton transfer step.

The agmatinase enzymes, which catalyze the hydrolysis of agmatine to putrescine, are key components of the polyamine biosynthetic pathway. These enzymes also appear to play a potential role in regulating neurotransmission in higher organisms by modulating agmatine concentrations. Because of their central importance in these processes, agmatinases and agmatinase-like proteins are emerging drug targets, both in microbes and in humans [5, 13, 24, 57–59]. Structural elucidation of the overall fold and key active site residues, as described here for SPEB from *E*. *coli*, provide important insights into substrate binding interactions and enzyme mechanism, both of which provide a solid foundation for future drug development efforts.

## Supporting information

S1 File (DOCX)
