# Supplementary material for: Structure of the E. coli agmatinase, SPEB
Source: PLoS One. 2021 Apr 15;16(4):e0248991. doi: 10.1371/journal.pone.0248991 (PMC8049259; doi:10.1371/journal.pone.0248991)
Supplement: S1 File — (DOCX) [file pone.0248991.s001.docx]

Structure of the *E. coli* Agmatinase, SPEB^†^

Iva Chitrakar^1,2^, Fardin Ahmed^3^, Andrew T. Torelli^3,^*, Jarrod B. French^1,4,5,^*^,‡^

1. Department of Biochemistry and Cell Biology, Stony Brook University, Stony Brook, NY 11794

2. Biochemistry and Structural Biology Graduate Program, Stony Brook University, Stony Brook, NY, 11794

3. Department of Chemistry, Ithaca College, Ithaca, NY 14850

4. Chemistry Department, Stony Brook University, Stony Brook, NY

5. Hormel Institute, University of Minnesota, Austin, MN 55912

* Corresponding Authors:

ATT: (607) 274-3576; [atorelli@ithaca.edu](mailto:atorelli@ithaca.edu)

JBF: (507) 437-9637; [jfrench@umn.edu](mailto:jfrench@umn.edu)

S1 Fig. Gene sequence used for SPEB

S2 Fig. Amino acid sequence of expressed protein

S3 Fig. SEC chromatogram of SPEB protein

S4 Fig. SDS PAGE gel showing expressed SPEB after purification

S5 Fig. Electron density at SPEB active site

S6 Fig. Structural comparison to *P. aeruginosa* guanidinopropionase (3NIP) and to *D. radiodurans* agmatinase (1WOG)

S7 Fig. Comparison of SPEB hexameric structure to *Schistosoma mansoni* trimer (4Q3V)

S8 Fig. Asymmetric unit of SPEB crystal.

S9 Fig. Sequence alignment of SPEB and homologues

CATATGTCAACCCTGGGCCATCAGTATGATAACTCGCTGGTGTCTAATGCTTTTGGCTTCCTGCGTCTGCCGATGAACTTCCAACCGTATGATAGCGACGCCGATTGGGTGATTACCGGCGTTCCGTTTGATATGGCTACGTCTGGTCGTGCGGGCGGTCGTCATGGTCCGGCAGCAATCCGTCAGGTGTCTACCAACCTGGCATGGGAACACAACCGCTTTCCGTGGAATTTCGATATGCGTGAACGCCTGAATGTGGTTGACTGCGGCGATCTGGTTTATGCCTTTGGTGATGCACGTGAAATGTCCGAAAAACTGCAGGCCCATGCAGAAAAACTGCTGGCAGCTGGCAAACGTATGCTGTCATTTGGCGGTGATCACTTCGTTACCCTGCCGCTGCTGCGCGCTCATGCGAAACACTTTGGTAAAATGGCCCTGGTCCATTTCGACGCACACACCGATACGTATGCTAACGGCTGTGAATTTGACCATGGTACCATGTTCTACACGGCGCCGAAAGAAGGCCTGATTGATCCGAATCACTCGGTGCAGATTGGCATCCGTACCGAATTTGACAAAGATAACGGTTTCACGGTTCTGGACGCATGCCAAGTCAATGATCGCAGCGTTGATGACGTCATTGCGCAGGTCAAACAAATCGTGGGTGACATGCCGGTTTACCTGACCTTTGACATTGATTGTCTGGATCCGGCATTCGCACCGGGTACCGGTACGCCGGTCATTGGCGGTCTGACGTCTGATCGTGCCATCAAACTGGTGCGCGGCCTGAAAGACCTGAACATCGTGGGTATGGATGTCGTGGAAGTTGCTCCGGCGTATGATCAGTCCGAAATTACCGCACTGGCAGCAGCAACGCTGGCACTGGAAATGCTGTACATCCAAGCTGCGAAAAAAGGTGAA**TAA**CTCGAG

**S1 Fig.** Gene sequence used for SPEB. Shown is the gene that was synthesized and inserted into the pTHT vector. The restriction endonuclease sites are underlined and the stop codon is bolded.

**MGSDKIHHHHHHSSGENLYFQGH**

MSTLGHQYDNSLVSNAFGFLRLPMNFQPYDSDADWVITGVPFDMATSGRAGGRHGPAAIR

QVSTNLAWEHNRFPWNFDMRERLNVVDCGDLVYAFGDAREMSEKLQAHAEKLLAAGKRML

SFGGDHFVTLPLLRAHAKHFGKMALVHFDAHTDTYANGCEFDHGTMFYTAPKEGLIDPNH

SVQIGIRTEFDKDNGFTVLDACQVNDRSVDDVIAQVKQIVGDMPVYLTFDIDCLDPAFAP

GTGTPVIGGLTSDRAIKLVRGLKDLNIVGMDVVEVAPAYDQSEITALAAATLALEMLYIQ

AAKKGE

**S2 Fig**. Amino acid sequence of expressed protein. The sequence of SPEB, as expressed, is shown. The leader sequence with the affinity tag, from the pTHT vector, is bolded. Note that the tag was not removed after purification.


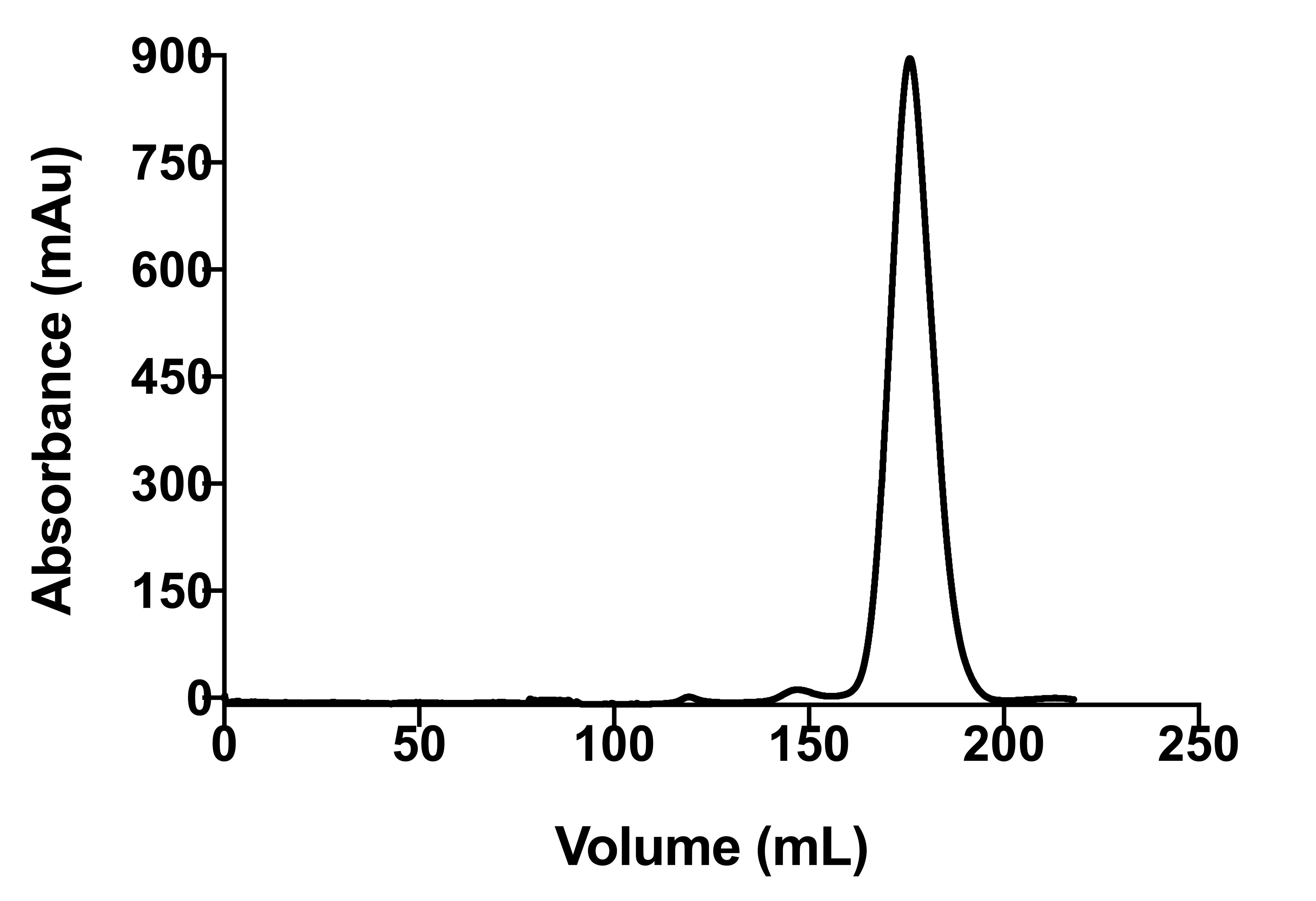


**S3 Fig**. SEC chromatogram of SPEB protein. Based on a comparison to a set of standards run under similar conditions (MW=440 kDa, V_e_=118 mL; MW=232 kDa, V_e_=148 mL; MW=158 kDa; V_e_=178 mL; MW=66 kDa, V_e_=192 mL), the V_e_ of SPEB (172 mL) suggests a molecular weight of ~160-170 kDa. This is consistent with 5 to 6 chains of SPEB.


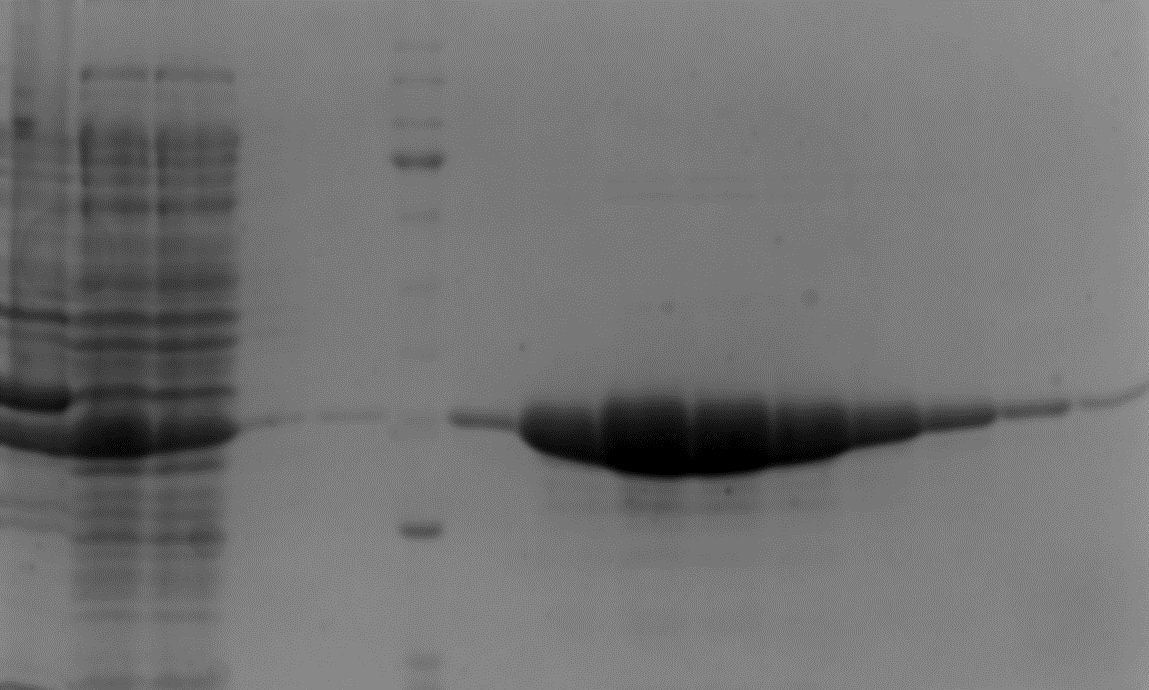


**L**

**25**

**85**

**50**

**30**

**40**

**S4 Fig**. SDS PAGE gel showing expressed SPEB after purification. Molecular weights (in kDa) are shown beside the bands for the molecular weight markers (lane marked with L). The final elution fractions of the purified protein are on the right side of the gel. The expected weight of the protein is approximately 33 kDa.


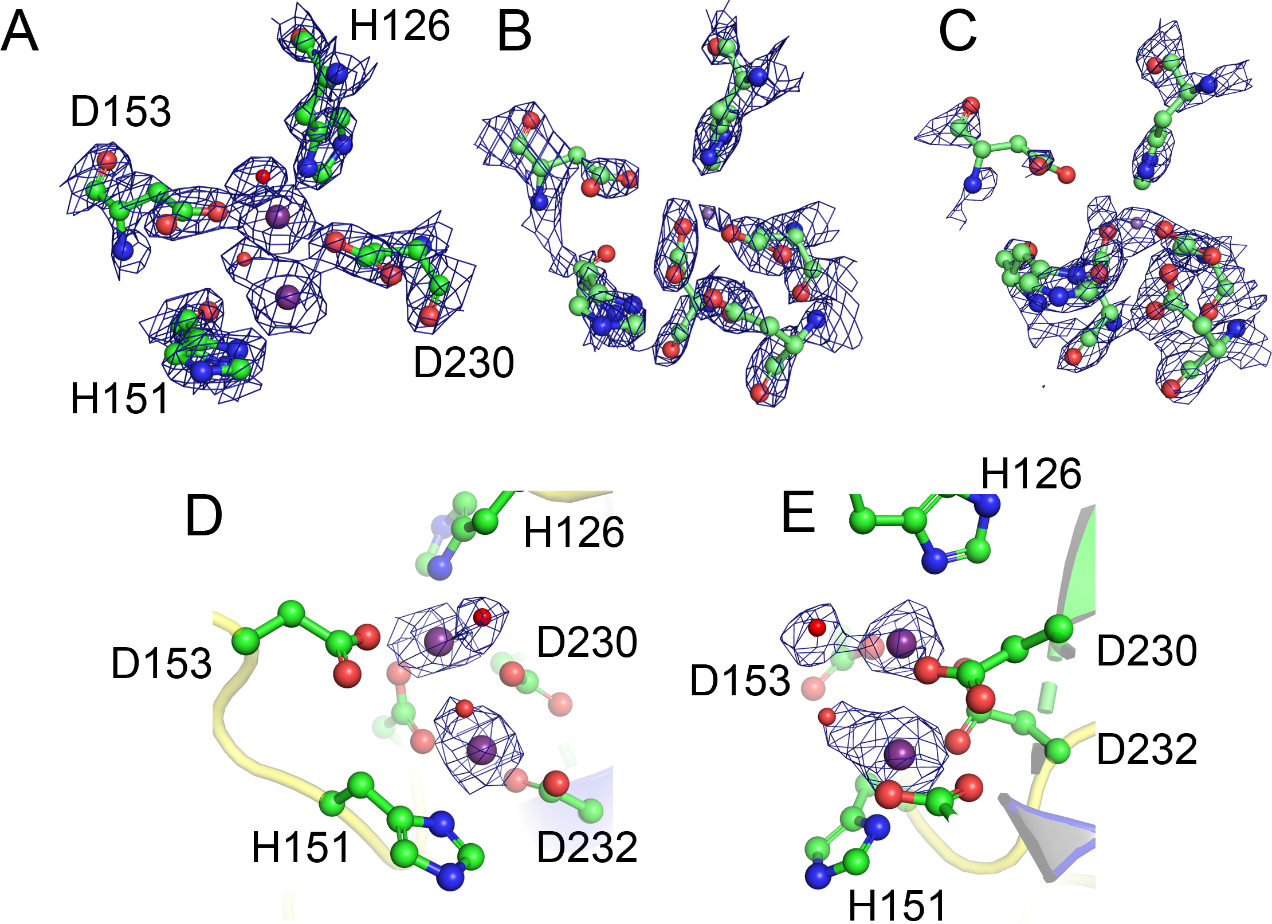


**S5 Fig**. Electron density at SPEB active site. Clear electron density around the active site in hexamers ABCDEF and GHIJKL allowed for the unambiguous placement of sidechains and all metals (A, density shown for chain A). Electron density was less clear in hexamer MNOPQR (B, density shown for chain M, and C, density shown for chain Q) and allowed the unambiguous of only one of the two metal ions in most of the chains within that hexamer. D and E show representative electron density for the metal ions and water molecules alone (chain A is shown). A, B, C and D are shown in a similar orientation as in Figure 5A (149 and D232 have been omitted in A for clarity), while E is rotated 90° relative to D. All of these maps are difference density maps, contoured at 2.5 σ, generated from structures where the residues, metals and water atoms of interest had been removed. The final model is shown superimposed over the difference maps. Atom coloring: carbon: green, nitrogen: blue, oxygen: red, Mn^2+^ ions: purple, peptide backbone: yellow.


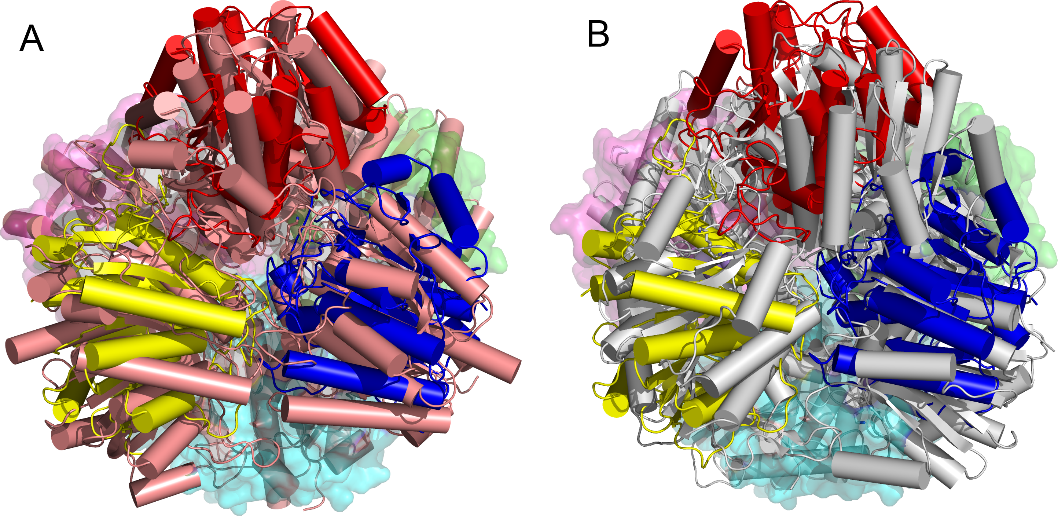


**S6 Fig.** Structural comparison to *Pseudomonas aeruginosa* guanidinopropionase (3NIP), and *Deinococcus radiodurans* agmatinase (1WOG). The overall orientation of the hexamer of *P. aeruginosa* guanidinopropionase (A, pink; RMSD 37.3 Å) and the hexamer of *D. radiodurans* agmatinase (B, grey; RMSD 29.0 Å) are similar to that of SPEB (top 3 chains shown in cartoon representation and colored with red, yellow and blue helices, respectively, while the bottom 3 chains are shown in surface representation and colored in cyan, green and light purple, respectively).

**
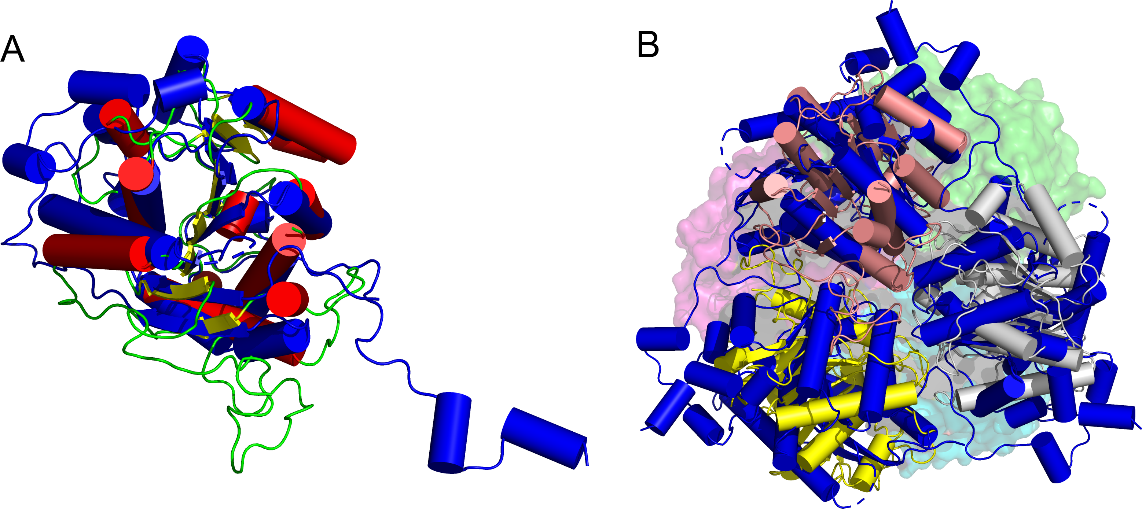
**

**S7 Fig.** Comparison of SPEB structure to *Schistosoma mansoni* agmatinase (4Q3V). The protomer of SPEB (A, red helices) superimposes well on the protomer of the *S. mansoni* agmatinase (A, blue; RMSD 1.4 Å). The organization of the *S. mansoni* trimer (B, blue) has similar symmetry as that of the SPEB (B), although the orientation of the chains with respect to one another is different (RMSD 4.6 Å).

**
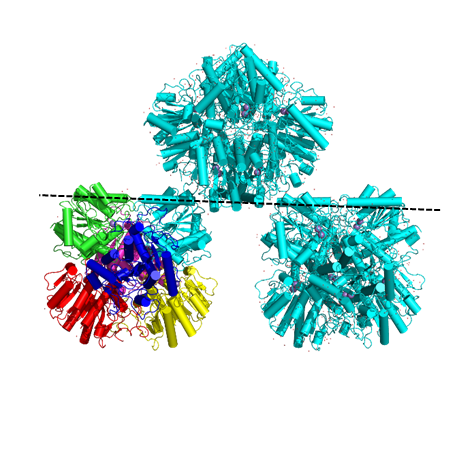
S8 Fig**. Asymmetric unit of SPEB crystal. After extensive analysis and accounting for twinning, the asymmetric unit of the SPEB crystal was determined to include 18 chains for a total of 3 hexamers. The dashed line drawn through the two helices at the top of the lower left hexamer of SPEB shows the subtle rotation of the lower right hexamer, with respect to the other.


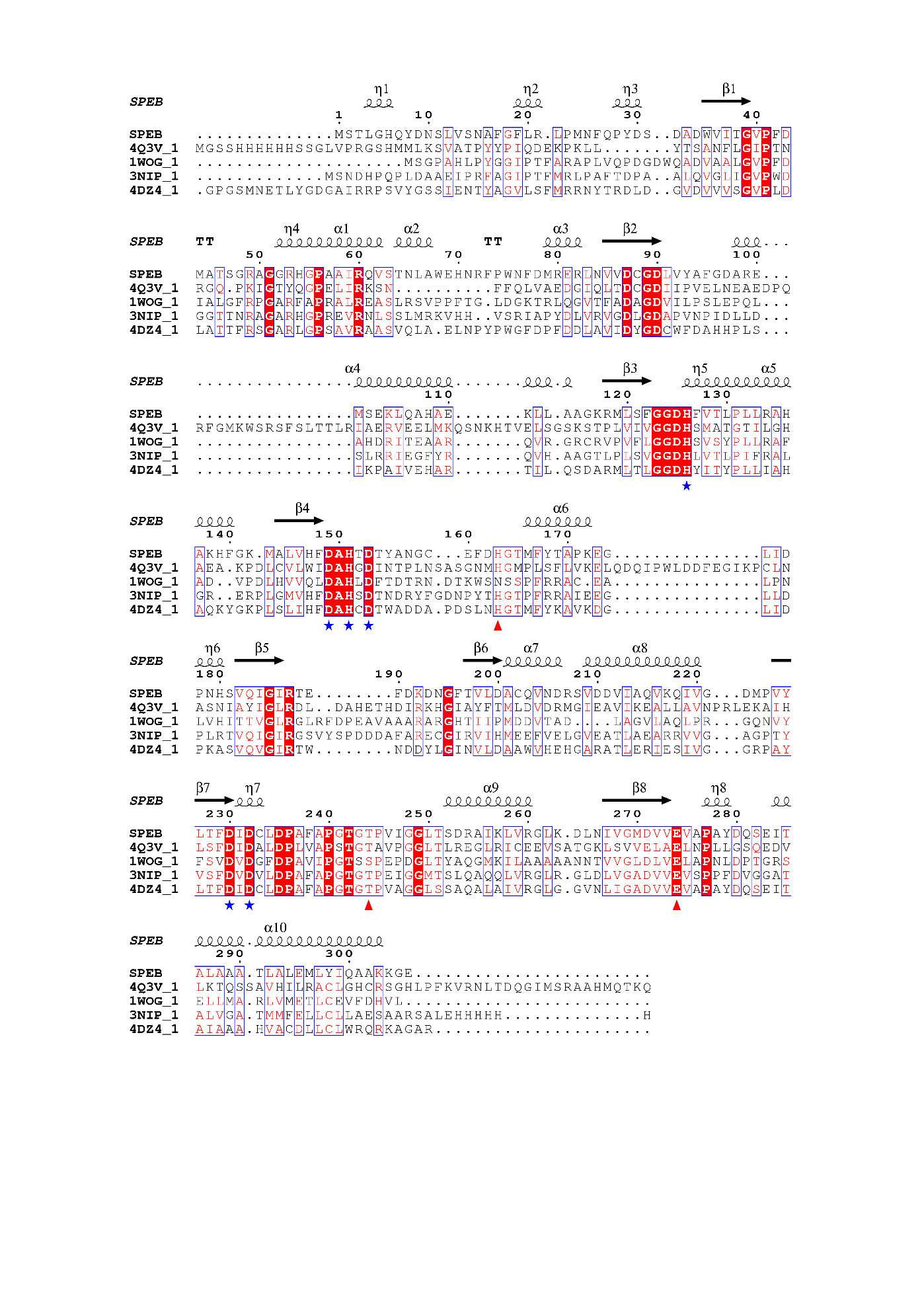
**S9 Fig**. Alignment of SPEB sequence with those of *S. mansoni* agmatinase (4Q3V), *D. radiodurans* agmatinase (1WOG), *P. aeruginosa* guanidinopropionase (3NIP), and *B. thailandensis* hypothetical agmatinase (4DZ4). The secondary structure elements shown are from SPEB. Blue stars mark the highly conserved metal-binding residues, while red triangles mark additional residues observed in the SPEB active site. This figure was generated using ESPRIPT (48).
